# Supplementary material for: A computational framework for a Lyapunov-enabled analysis of biochemical reaction networks
Source: PLoS Comput Biol. 2020 Feb 24;16(2):e1007681. doi: 10.1371/journal.pcbi.1007681 (PMC7058358; doi:10.1371/journal.pcbi.1007681)
Supplement: S1 Text — (PDF) [file pcbi.1007681.s001.pdf]

# Supplementary Information to "A Lyapunov-enabled analysis of biochemical reaction networks"

M. Ali Al-Radhawi[1], David Angeli[1,3], and Eduardo D. Sontag[1, 4]

[1]Departments of Bioengineering and of Electrical and Computer Engineering, Northeastern University, Boston, MA 02115.
[2]Department of Electrical & Electronic Engineering, Imperial College London, London SW7 2AZ, UK.
[3]Dipartimento di Ingegneria dell'Informazione, University of Florence, Florence, Italy.

## 1 Lyapunov's Second Method

### 1.1 Preliminaries

First, let consider *specific* kinetics $R \in \mathscr{K}_A$. Hence, the ODE is given as

$$\dot{x} = f(x) := \Gamma R(x). \tag{1}$$

We have the following definition:

**Definition A-1.** *Given the ODE* (1)*. Let* $V : \mathbb{R}^n_{\geq 0} \to \mathbb{R}_{\geq 0}$ *be locally Lipschitz. Then* $V$ *is said to be a Lyapunov function for* (1) *if* $V$ *is*

- Positive-Definite *(with respect to the steady states set) if* $V(x) \geq 0$*, and* $V(x) = 0$ *if and only if* $R(x) \in \ker \Gamma$.
- Nonincreasing *if* $\dot{V}(x) := D^+_f V(x) \leq 0$ *for all* $x$*, where* $D^+_f$ *is defined below.*

Note that when $\partial V/\partial x$ exists at a point $x$, then $\dot{V}(x) = \partial V/\partial x \Gamma R(x)$.

#### 1.1.1 Generalized Derivatives

The function $V$ is locally Lipschitz, and hence it is not necessarily differentiable everywhere. It has been known since the early stability literature (see [1], [2]) that the standard Lyapunov theorems can be generalized without difficulty with locally Lipschitz Lyapunov functions and Dini derivatives.

The upper Dini derivative for $V$ in the direction of a function $f(x) := \Gamma R(x)$ is defined as:

$$D^+_f V(x) := \limsup_{h \to 0^+} \frac{V(x + h\Gamma R(x)) - V(x)}{h}. \tag{2}$$

For a locally Lipschitz function, the above quantity is always finite.

An alternative definition of the derivative, which is more restrictive but has more convenient calculus, is the *Clarke derivative*, which is defined as [3]:

$$D^C_f V(x(t)) := \limsup_{\substack{h \to 0^+ \\ y \to x}} \frac{V(y + h\Gamma R(x)) - V(y)}{h}. \tag{3}$$

Note that $D^+_f(x)V(x) \leq D^C_f V(x(t))$. We will define $\dot{V}$ in the sense of Dini.

### 1.1.2 LaSalle's Condition

Conventional stability theory [1] examines stability with respect to an isolated steady state. However, for reaction networks, there is usually a *continuum* of equilibria. This means that asymptotic stability or Lyapunov stability are not achieved in the classical sense. Nevertheless, the state space of reaction networks is divided into stoichiometric compatibility classes which are forward invariant. Furthermore, a stoichiometric class can sometimes be divided into *kinetic compatibility classes* [4]. In general, any initial condition $x_\circ$ is associated with a compatibility class $\mathscr{C}_{x_0}$. Hence we state the following definition:

**Definition A-2** (The LaSalle's Condition)**.** *Given an ODE* (1) *with a Lyapunov function $V$. The* LaSalle's Condition *is satisfied if the following statement holds:*
*If a solution $\varphi(t; x_\circ)$ of* (1) *satisfies $\varphi(t; x_\circ) \in \ker \dot{V} \cap \mathscr{C}_{x_\circ}$ for all $t \geq 0$, then $\varphi(t; x_\circ) \in E_{x_\circ}$ for all $t \geq 0$, where $E_{x_\circ} \subset \mathscr{C}_{x_\circ}$ is the set of steady states for* (1) *contained in $\mathscr{C}_{x_\circ}$.*

### 1.1.3 Lyapunov Stability Theorem

We state the following theorem which is standard Lyapunov theory adapted to our settings [5].

**Theorem A-1** (Lyapunov's Second Method)**.** *Given* (1) *with initial condition $x_\circ \in \mathbb{R}^n_+$. Let $\mathscr{C}_{x_\circ}$ be its class. Assume there exists a Lyapunov function $V$ and suppose that $x(t)$ is bounded.*

- *Then the steady state set $E_{x_\circ}$ is Lyapunov stable relative to $\mathscr{C}_{x_\circ}$.*
- *If, in addition, the LaSalle's Condition is satisfied, then $x(t) \to E_{x_\circ}$ as $t \to \infty$ (i.e., the point to set distance of $x(t)$ to $E_{x_\circ}$ tends to 0). Furthermore, any isolated steady state relative to $\mathscr{C}_{x_\circ}$ is asymptotically stable.*
- *If the LaSalle's condition is satisfied, and all the trajectories are bounded, then: if there exists an $x^* \in E_{x_\circ}$ which is isolated relative to $\mathscr{C}_{x_\circ}$, then it is unique, i.e., $E_{x_\circ} = \{x^*\}$. Furthermore, it is globally asymptotically stable steady state relative to $\mathscr{C}_{x_\circ}$.*

## 1.2 Robust Lyapunov Functions and Proof of Theorem 1

In the main text, we have defined an RLF $\tilde{V} : \mathbb{R}^\nu \to \mathbb{R}_{\geq 0}$. For a given $R \in \mathscr{K}_A$, the Lyapunov function is $V(x) = \tilde{V}(R(x))$.

Before proving Theorem 1, we need to state and prove the following Lemma:

**Lemma A-1.** *Let $\dot{x} := f(x)$, and let $V : \mathbb{R}^n_{\geq 0} \to \mathbb{R}_{\geq 0}$ be a locally Lipschitz function such that:*

$$\frac{\partial V(x)}{\partial x} f(x) \leq 0 \text{ whenever } \frac{\partial V(x)}{\partial x} \text{ exists},$$

*Then $\dot{V}(x) \leq 0$ for all $x$.*

*Proof.* Since $V$ is assumed to be locally Lipschitz, Rademacher's Theorem implies that it is differentiable (i.e., gradient exists) almost everywhere [3]. Recall that for a locally Lipschitz function the *Clarke gradient* at $x$ is defined as $\partial_C V(x) := \operatorname{co} \partial V(x)$, where:

$$\partial V(x) := \left\{ p \in \mathbb{R}^n : \exists x_i \to x \,\text{with}\, \partial V(x_i)/\partial x \text{ exists, such that,} \, p = \lim_{i \to \infty} \partial V(x_i)/\partial x \right\}.$$

Let $p \in \partial V(x)$ and let $\{x_i\}_{i=1}^{\infty}$ be any sequence as in the definition of the Clarke gradient. By the assumption stated in the Lemma, $(\partial V(x_i)/\partial x) f(x_i) \leq 0$, for all $i$. Hence, the definition of $p$ implies that $p^T f(x) \leq 0$. Since $p$ was arbitrary, the inequality holds for all $p \in \partial V(x)$.
Now, let $p \in \bar{\partial} V(x)$ where $p = \sum_i \lambda_i p_i$ is a convex combination of any $p_1, ..., p_{n+1} \in \partial V(x)$. By the inequality above, $p^T f(x) = \sum_i \lambda_i (p_i^T f(x)) \leq 0$. Hence, $p^T f(x) \leq 0$ for all $p \in \bar{\partial} V(x)$.
As in [3], the Clarke derivative of $V$ at $x$ in the direction of $f(x)$ can be written as $D^C_{f(x)} V(x) = \max\{p^T f(x) : p \in \bar{\partial} V(x)\}$. By the above inequality, we get $D^C_{f(x)} V(x) \leq 0$ for all $x$. Since the Dini derivative is upper bounded by the Clarke derivative, we finally get:

$$\dot{V}(x) := \limsup_{h \to 0^+} \frac{V(x + hf(x)) - V(x)}{h} \leq \limsup_{\substack{h \to 0^+ \\ y \to x}} \frac{V(y + hf(x)) - V(y)}{h} =: D^C_{f(x)} V(x) \leq 0,$$

for all $x$. □

**Proof of Theorem 1** We show that the existence of the common Lyapunov function implies the existence of the RLF. Nonnegativity of $V$ follows from the nonnegativity of $\tilde{V}$. Let $(i, j) = \kappa(\ell)$, and recall that $Q_\ell = e_j \gamma_i^T$, hence $\ker \tilde{V} = \bigcap_{\ell=1}^s \ker Q_\ell = \ker \Gamma$. Therefore, $R(x) \in \ker V$ iff $\Gamma R(x) = 0$, which establishes the positive-definiteness of $V$.

We assumed that $\tilde{V}$ has a negative semi-definite time-derivative for every linear system in the considered set. Hence, whenever $\tilde{V}$ is differentiable at a point $r$, we can write $(\partial \tilde{V}/\partial r) Q_\ell r \leq 0$, $\ell = 1, ..., s$. Hence, for any $\rho^1, ..., \rho^s \in \bar{\mathbb{R}}_+$:

$$\sum_{\ell=1}^{s} \rho^\ell \frac{\partial \tilde{V}}{\partial r} Q_\ell r \leq 0, \text{whenever} \, (\partial V(r)/\partial r) \text{ exists.} \tag{4}$$

Therefore, whenever $\tilde{V}$ is differentiable we have

$$\dot{V}(x) = \frac{\partial \tilde{V}}{\partial R} \frac{\partial R}{\partial x}(x) \Gamma R(x) = \frac{\partial \tilde{V}}{\partial R} \left( \sum_{i,j:\alpha_{ij}>0} \frac{\partial R_j}{\partial x_i}(x) E_{ji} \right) \Gamma R(x) \tag{5}$$

where $\partial \tilde{V}/\partial R := (\partial \tilde{V}/\partial r)\Big|_{r=R(x)}$.
Now, denote $\rho^\ell = \frac{\partial R_j}{\partial x_i}(x)$, which is nonnegative by AK3. This allows us to write:

$$\dot{V}(x) = \sum_{\ell=1}^{s} \rho^\ell \frac{\partial \tilde{V}}{\partial R} E_{ji} \Gamma R(x) \tag{6}$$

$$= \sum_{\ell=1}^{s} \rho^\ell \frac{\partial \tilde{V}}{\partial R} Q_\ell R(x) \leq 0, \text{ for almost all} \, x. \tag{7}$$

The last inequality follows from (4). Using Lemma A-1, $\dot{V}(x) \leq 0$ for all $x$, and for all $R \in \mathscr{K}_A$.

In order to show the other direction, since most of the properties outlined in the RLF definition are clearly satisfied, it remains to show nonincreasingness. Assume that there exists $\ell$ such that $\tilde{V}(r)$ is not nonincreasing along the trajectories of $\dot{r} = Q_\ell r$. Consider the corresponding term in (6). Since $V(R(x))$ is a Lyapunov function for any choice of admissible rate reaction function $R$, choose $\rho^\ell = \frac{\partial R_j}{\partial x_i}$ to be large enough such that $\dot{V}(x) \geq 0$ for some $x$; this results in a contradiction.

## 2 Piecewise Linear RLFs

### 2.1 Checking a candidate Lyapunov function

Suppose we are given a matrix $H \in \mathbb{R}^{p\times r}$ such that $\ker H = \ker \Gamma$. Let $\tilde{V} : \mathbb{R}^\nu \to \mathbb{R}$ be a continuous PWL function given as

$$\tilde{V}(r) = |c_k^T r|, \; r \in \pm\mathcal{W}_k, k = 1, .., m/2,$$

where the regions $\mathcal{W}_k = \{r \in \mathbb{R}^\nu : \Sigma_k H r \geq 0\}, k = 1, .., m$ form a proper conic partition of $\mathbb{R}^\nu$, while $\{\Sigma_k\}_{k=1}^m$ are signature matrices (diagonal matrices with $\pm 1$ on the diagonal) with the property $\Sigma_k = -\Sigma_{m+1-k}, k = 1, .., m/2$. (see [5] for a detailed exposition on the geometry of the partition regions).

The coefficient vectors of each linear component can be collected in a matrix $C = [c_1, .., c_{\frac{m}{2}}]^T \in \mathbb{R}^{\frac{m}{2}\times r}$. If the function $\tilde{V}$ is convex, then we have the following simplified representation of $V$ [6]:

$$V(x) = \|CR(x)\|_\infty.$$

This representation is analogous of the $\ell_\infty$-norm Lyapunov functions that have been used for linear systems in [7].

**Theorem A-2.** *Let $\Gamma$ and $H$ be given as above. Let $\tilde{V}$ be a candidate continuous nonnegative PWL function with $C = [c_1 \; ... \; c_{\frac{m}{2}}]^T \in \mathbb{R}^{\frac{m}{2}\times r}$. Then $\tilde{V}$ is an RLF if and only if:*

- $\ker C = \ker \Gamma$, *and*
- *there exists matrices $\{\Lambda^\ell\}_{\ell=1}^s \subset \mathbb{R}^{\frac{m}{2}\times\frac{m}{2}}$ such that*

$$\Lambda^\ell H = -CQ_\ell, \tag{8}$$

*and $\lambda_k^\ell \Sigma_k > 0$, where $\Lambda^\ell = [\lambda_1^{\ell\,T} ... \lambda_{m/2}^{\ell\;\,T}]^T$.*

*If $\tilde{V}$ is convex, then the second condition can be replaced with*

*2) there exists Metzler matrices $\{\Lambda^\ell\}_{\ell=1}^s \subset \mathbb{R}^{m\times m}$ such that*

$$\Lambda^\ell \tilde{C} = \tilde{C} Q_\ell, \tag{9}$$

*and $\Lambda^\ell \mathbf{1} = 0$ for all $\ell = 1, .., s$, where $\tilde{C} = [C^T \;\; -C^T]^T$.*

*Proof.* The proof can be carried out by performing algebraic manipulations on the results presented in [5, Theorem 4]. The function has been assumed continuous and nonnegative. It remains to show that the corresponding condition in [5] is equivalent to (9). Considering (9) row by row, it can be written in the following form for $k \in \{1, .., \frac{m}{2}\}$:

$$\lambda_k^{\ell\,T} H = -c_{kj}\alpha_{ij}\gamma_i^T,$$

where $(i, j) = \kappa(\ell)$. $c_{kj}$ can be replaced with $\mathrm{sgn}(c_{kj})$ and $\alpha_{ij}$ can be replaced with 1 if we are considering only $i \in I_k, j \in J_{ki}$. Therefore, equivalence with the corresponding condition in [5, Theorem 4] is established.

For a convex PWL function, we can write the corresponding condition in [5, Theorem 5] as follows after replacing $\mathrm{sgn}(c_{kj})$ by $c_{kj}$, and inserting $\alpha_{ij}$:

$$-c_{kj}\alpha_{ij}\gamma_i^T = \left(\sum_{j=1, j\neq k}^m \lambda_{kj}^\ell\right) c_k^T - \sum_{j=1, j\neq k}^m \lambda_{kj}^\ell c_j^T.$$

Let $\lambda_{kk} = \sum_{j=1,j\neq k}^{m} \lambda_{kj}^{\ell}$. Then

$$c_k^T e_j \gamma_i^T = \sum_{j=1,j\neq k}^{m} \lambda_{kj}^{\ell} c_j^T - \lambda_{kk}^{\ell} c_k^T,$$

which enforces $\Lambda^{\ell}$ to be Metzler and $\Lambda^{\ell}\mathbf{1} = 0$ as above. □

**Remark A-1.** *The symmetries in equation* (9) *imply that it can be written equivalently as:*

$$CQ_{\ell} = \tilde{\Lambda}^{\ell} C, \tag{10}$$

*where $\tilde{\Lambda}^{\ell}$ is an $\frac{m}{2} \times \frac{m}{2}$ matrix which is defined by subtracting the upper $\frac{m}{2} \times \frac{m}{2}$ blocks of $\Lambda^{\ell}$ from each other. The matrix $\tilde{\Lambda}^{\ell}$ satisfies:*

$$\max_k \left( \tilde{\lambda}_{kk}^{(\ell)} + \sum_{j\neq k} |\tilde{\lambda}_{kj}^{(\ell)}| \right) \leq 0. \tag{11}$$

*This is exactly the condition that $\ell_\infty$-norm Lyapunov functions need to satisfy for a linear system [8, 9]. This shows that Theorem 1 provides the framework to utilize the existing linear stability analysis techniques in the literature to construct robust Lyapunov functions for nonlinear systems such as CRNs. For example, we can verify $\ell_1$ Lyapunov functions of the form $V(x) = \|CR(x)\|_1$ directly by replacing condition* (11) *by*

$$\max_k \left( \tilde{\lambda}_{kk}^{(\ell)} + \sum_{j\neq k} |\tilde{\lambda}_{jk}^{(\ell)}| \right) \leq 0, \tag{12}$$

*instead of converting them to the $\ell_\infty$-norm form.*

## 2.2 Proof of Theorem 2

The linear program has the parametrization $c_k^T = \xi_k^T \Sigma_k H$, which follows from applying Farkas's Lemma to ensure that $\tilde{V}(r) = c_k^T r \geq 0$ on the region $\mathcal{W}_k$. (See [5] for full details.) The second condition: $CQ_{\ell} = -Q_{\ell} H$ follows from Theorem 2 and ensures that $V(R(x))$ is nonincreasing. To ensure continuity we need to have $c_k^T r = c_{\ell}^T r$ whenever $r \in \mathcal{W}_k \cap \mathcal{W}_{\ell}$. Since $\ker H = \ker \Gamma$, continuity can be imposed by the constraint $(c_k - c_{\ell})^T v_i = 0$ for $i = 1, .., \dim(\ker \Gamma)$. □

## 2.3 Enforcing convexity in a linear program

The linear program presented in the main text does not enforce convexity on the PWL RLF. Following [5], we describe how to write a linear program to construct convex PWL RLFs in what follows.

We need to introduce the concept of a neighbor to a region. Fix $k \in \{1, .., m/2\}$. Consider the matrix $H$: for any pair of linearly dependent rows $h_{i_1}^T, h_{i_2}^T$ eliminate $h_{i_2}^T$. Denote the resulting matrix by $\tilde{H} \in \mathbb{R}^{\tilde{p}\times\nu}$, and let $\tilde{\Sigma}_1, .., \tilde{\Sigma}_m$ the corresponding signature matrices. Therefore, the region can be represented as $\mathcal{W}_k = \{r | \tilde{\Sigma}_k \tilde{H} r \geq 0\}$. The *distance* $d_r$ between two regions $\mathcal{W}_k, \mathcal{W}_j$ is defined to be the *Hamming distance* between $\tilde{\Sigma}_k$ and $\tilde{\Sigma}_j$. Hence, the set of neighbors of a region $\mathcal{W}_k$ are defined as:

$$\mathcal{N}_k = \{j \in \{1, 2, \ldots, m\} : d_r(\mathcal{W}_j, \mathcal{W}_k) = 1\}.$$

Equivalently, note that a neighboring region to $\mathcal{W}_k$ is one which differs only by the switching of one inequality. Denote the index of the switched inequality by the map $s_k(.) : \mathcal{N}_k \to \{1, .., p\}$. For simplicity, we use the notation $s_{k\ell} := s_k(\ell)$.

**Theorem A-3.** *Given the system* (1) *and a partitioning matrix* $H \in \mathbb{R}^{p\times r}$*. Consider the linear program:*

$$\begin{aligned}
\textit{Find} \quad & c_k, \xi_k, \zeta_k \in \mathbb{R}^{\nu}, \Lambda^{\ell} \in \mathbb{R}^{m\times m}, \eta_{kj} \in \mathbb{R}, \\
& k = 1, .., \tfrac{m}{2}; j \in \mathcal{N}_k, \ell = 1, .., s, \\
\textit{subject to} \quad & c_k^T = \xi_k^T \Sigma_k H, \\
& CQ_{\ell} = -\Lambda^{\ell} H, \lambda_k^{\ell} \Sigma_k \geq 0, \\
& c_k - c_j = \eta_{kj} \sigma_{k s_{kj}} h_{s_{kj}}, \\
& \xi_k \geq 0, \mathbf{1}^T \xi_k > 0, \Lambda^{\ell} \geq 0,
\end{aligned}$$

*where* $\sigma_{kj}$ *is the* $j$*th entry on the diagonal of* $\Sigma_k$*. Then there exists a PWL RLF with partitioning matrix* $H$ *if and only if there exists a feasible solution to the above linear program that satisfies* $\ker C = \ker \Gamma$*. Furthermore, the PWL RLF can be made convex by adding the constraints* $\eta_{kj} \geq 0$*.*

## 2.4 Networks without positive steady states

Let AS1 be the assumption that requires the existence of a positive vector in $\ker \Gamma$, which is a necessary condition for the existence of positive steady states. This assumption simplifies the geometry of the partition regions and enforces symmetry on the coefficient matrix $C$. Nevertheless, our techniques can be extended without difficulty for the construction of PWL RLFs for generic CRNs that do not satisfy AS1. Consider a matrix $H \in \mathbb{R}^{p\times \nu}$, with $\ker H = \ker \Gamma$. The regions are defined as:

$$\mathcal{W}_k = \{r \in \mathbb{R}^{\nu} : \Sigma_k H r \geq 0, r \geq 0\},$$

where $k = 1, .., 2^p$. Note that the inequality $r \geq 0$ needs to be explicitly included. As before, let $m$ be the number of non-empty interior regions. Then, the regions are ordered such that the first $m$ regions are the non-empty interior ones. Therefore, the following theorem can be stated for networks that do not necessarily satisfy AS1.

**Theorem A-4.** *Consider the system* (1)*, with* $H = [\Gamma^T \hat{H}^T]^T$*,* $\{\Sigma_k\}_{k=1}^m$ *given as before. Consider the following linear program:*

$$\begin{aligned}
\textit{Find} \quad & c_k, e_k, \xi_k, \zeta_k \in \mathbb{R}^{\nu}, \Lambda^{\ell} = [\lambda_1^{\ell\,T} ... \lambda_m^{\ell\,T}]^T \in \mathbb{R}^{m\times m} \\
& k = 1, .., m; \ell = 1, .., s, j = k+1, .., m \\
\textit{subject to} \quad & c_k^T = \xi_k^T \Sigma_k H + e_k^T, \\
& CQ_{\ell} = -\Lambda^{\ell} H, \lambda_k^{\ell} \Sigma_k \geq 0, \\
& (c_k - c_j)^T v_i = 0, i = 1, .., \dim(\ker \Gamma) \\
& \xi_k \geq 0, \mathbf{1}^T \xi_k > 0, e_k \geq 0 \Lambda^{\ell} \geq 0,
\end{aligned}$$

*Then there exists a PWL RLF with partitioning matrix* $H$ *if and only if there exists a feasible solution to the above linear program with* $\ker C = \ker \Gamma$ *satisfied.*

### 2.5 Proof of Theorem 3

The algorithm starts with $C = \Gamma$. Hence, it can be interpreted as an initial PWL function $\tilde{V}(r) = \max_{k=0,1,..,n} c_k^T r$ where $c_k = \gamma_k, k = 1,..,n$.

We aim at restricting the *active* region of each function $c_k^T R(x(t))$ to the region on which it is nonincreasing, i.e $c_k^T \dot{R}(x(t)) \le 0$. This is accomplished by adding extra linear components that ensures this. Define the *active region* of a vector $c_k$, $k = 1,..,m_0$, as:

$$\mathcal{W}_0(c_k) := \{r \in \mathbb{R}^\nu : c_k^T r \ge c_j^T r, j \ne k\}.$$

We define the *permissible region* of a linear component $c_k$ to be the region for which it is nonincreasing:

$$\mathcal{P}(c_k) := \{r \in \mathbb{R}^\nu : \operatorname{sgn}(c_{kj})\gamma_i^T r \le 0 \text{ for all } (i,j) \text{ such that } j \in \operatorname{supp}(c_k) \text{ and } i \in \mathcal{I}(\mathbf{R}_j)\}.$$

Note that in general, $\mathcal{W}_0(c_k) \not\subset \mathcal{P}(c_k)$. Therefore, the iterative procedure defines a new PWL function with matrix $C_1$ so that $\mathcal{W}_1(c_k) \subset \mathcal{P}(c_k)$. To achieve this, new rows are added to $C$ as follows:

$$c_{m_0+i} := c_k + \operatorname{sgn}(c_{kj})\gamma_i \tag{13}$$

for all $(i,j)$ such that $j \in \operatorname{supp}(c_k)$ and $i \in \mathcal{I}(\mathbf{R}_j)$.

The procedure is repeated for every row of $C$. If the procedure terminates, i.e no new rows need to be added, then $\tilde{V}(r) = \max_{k=0,1,..,n} c_k^T r$ is a PWL RLF. □

# 3 Construction of PWQ RLFs

### 3.1 Proof of Theorem 5

We show that $\tilde{V}$ is a common Lyapunov function for $\{\dot{r} = Q_1 r, ..., \dot{r} = Q_s r\}$ as in Theorem 1.
In order to show nonnegativity, inequality (23) implies that:

$$r^T P_k r + 2c_k^T r \ge (\Sigma_k H r)^T (A_k^1 + A_k^2)\Sigma_k H r + 2c_k^T(\Sigma_k H) r,$$

and since $A_k^1 + A_k^2$ is copositive this implies that $\tilde{V}(r) \ge 0$ when $r \in \pm W_k, k = 1,.., \frac{m}{2}$, which establishes nonnegativity. Positive-definiteness follows from (26) and the assumption in the statement of the theorem.

For continuity, it is sufficient to establish it between neighboring regions. Therefore, assume $j \in \mathcal{N}_k$, and let $h_{s_k j}^T r = 0$ be the intersection hypersurface, then (25) implies that $r^T P_k r + 2c_k^T r = r^T P_j r + 2c_j^T r$ when $r \in \mathcal{W}_k \cap \mathcal{W}_j$. The other direction holds also by writing $P_k - P_j$ over the decomposition $\mathbb{R}^\nu = \operatorname{span}\{h_{s_{kj}}\} \oplus \operatorname{span}\{h_{s_{kj}}\}^\perp$. Continuity of $\tilde{V}$ implies also that $\tilde{V}$ is locally Lipschitz.

In order to show that this derivative is negative semi-definite, consider the $\ell^{\text{th}}$ system, and let $r \in \mathcal{W}_k^\circ$, then:

$$\dot{\tilde{V}}_\ell(r) = r^T(Q_\ell P_k + P_k Q_\ell) r + 2c_k^T Q_\ell r.$$

Note that (24) implies that $\dot{\tilde{V}}_\ell(r) \le 0$ when $r \in \mathcal{W}_k$. As it is true for all $k$, then $\dot{\tilde{V}}_\ell(r) \le 0$ for all $\ell = 1,..s$, and all r such that $\partial\tilde{V}(r)/\partial r$ exists. By Lemma 1, this implies that $\dot{\tilde{V}}_\ell(r) \le 0$ for $\ell = 1,..,s$. □

### 3.2 Proof of Proposition 6

Assume that $C \in \mathbb{R}^{\frac{m}{2}\times r}$ is given such that $\tilde{V}_L$ is a PWL RLF, where $\tilde{V}_L$ is defined as in (2.1). Let $\tilde{V}_Q$ be defined by

$$\tilde{V}_Q(r) = r^T P_k r = r^T \tilde{c}_k \tilde{c}_k^T r, r \in \mathcal{W}_k, k = 1, .., m.$$

The constraints (25),(26) are clearly satisfied. The inequality (23) is satisfied with $A_k^1 = A_k^2 = 0$, $k = 1, .., \frac{m}{2}$. It remains to show that (24) is satisfied.

Fix $\ell \in \{1, .., s\}$, $k \in \{1, .., \frac{m}{2}\}$. Then

$$-\dot{\tilde{V}}_\ell = -r^T(Q_\ell P_k + P_k Q_\ell)r = -r^T(Q_\ell \tilde{c}_k^T \tilde{c}_k + \tilde{c}_k \tilde{c}_k^T Q_\ell)r.$$

Since it is assumed that $\tilde{V}_L$ is PWL RLF, there exist $\lambda_k^\ell, \xi_k \geq 0$ such that $\tilde{c}_k^T = \xi_k^T \Sigma_k H$, $\tilde{c}_k^T Q_\ell = \lambda_k^\ell \Sigma_k H$. Therefore:

$$-\dot{\tilde{V}}_\ell = (\Sigma_k H r)^T (\lambda_k^\ell \xi_k + \xi_k {\lambda_k^\ell}^T)(\Sigma_k H r).$$

Hence, (24) is satisfied with $B_{k\ell}^1 = \lambda_k^\ell \xi_k + \xi_k {\lambda_k^\ell}^T, B_{k\ell}^2 = 0$. □

# 4 Properties of Attractive Networks

## 4.1 Robust non-degeneracy

A point $x_e$ of (1) is *non-degenerate* if the Jacobian evaluated at $x_e$ relative to $\mathscr{C}_{x_e}$ is nonsingular. More precisely, let us change coordinates using a transformation matrix $T = [T_1^T \ D]^T$, where $D^T$ has full row rank and $D^T\Gamma = 0$, and $T_1$ is any matrix such that $T$ is nonsingular. Then, the Jacobian in the new coordinates can be written as:

$$T\Gamma \frac{\partial R}{\partial x} T^{-1} = \begin{bmatrix} J_1 & J_2 \\ 0 & 0 \end{bmatrix}. \qquad (14)$$

Therefore, $x_e$ is nondegenerate iff $J_1$ evaluated at $x_e$ is nonsingular. The matrix $J_1$ is called a *reduced Jacobian.*

### 4.1.1 Proof of Theorem 7

Recall that for an attractive network with PWL RLF, the negative Jacobian is $P_0$ for any choice of $R \in \mathscr{K}_A$ [5]. Using the Cauchy-Binet formula [10], let $I \subset \{1, .., n\}$ be an arbitrary subset so that $|I| = k$. The corresponding principal minor can be written as:

$$\det{}_I\left(-\Gamma\frac{\partial R}{\partial x}\right) = \sum_{J\subset\{1,..,\nu\},|J|=k} \det(-\Gamma_{IJ}) \det\left(\frac{\partial R}{\partial x}_{JI}\right) = \sum_\iota a_\iota \prod_{\ell\in L_\iota\subset\{1,..,s\}} \rho_\ell,$$

where the last equality refers to the fact that the sum can be expressed as a linear combination of products of $\rho_1, ..., \rho_s$. We claim that the coefficients $a_\iota$ are all nonnegative. To show this, assume for the sake of contradiction that there is some negative $a_{\iota_*}$. If we set all $\rho$'s to zero except the ones appearing in the $\iota_*^{\text{th}}$ term, then this implies that the corresponding principal minor can be negative; a contradiction.

Now, the theorem can be proven by noting that the reduced Jacobian is non-singular iff the sum of all $k \times k$ principal minors of the negative Jacobian is positive, where $k = \operatorname{rank}(\Gamma)$. Since it is assumed that there exists a point for which the reduced Jacobian is non-singular, this implies that the sum of principal minors is positive for some choice of $\rho_1, .., \rho_s$. Since all of the principal minors are nonnegative, then at least one of them is positive. By AK4, that principal minor stays positive for any choice of positive $\rho_1, .., \rho_s$, i.e. it stays positive over the interior of $\mathbb{R}_+^n$. □

### 4.1.2 Uniqueness of the steady states

The following directly from Theorem 7.

**Proposition A-5.** *Consider a network* $(\mathscr{S}, \mathscr{R})$ *that satisfies AS1 and admits a PWL RLF. If there exists a non-degenerate positive steady state* $x_e$*, relative to* $\mathscr{C}_{x_e}$*, then it is unique.*

*Proof.* Theorem 7 has shown that the existence of an non-degenerate positive steady state $x_e$ ensures that the reduced Jacobian is non-singular on the interior of the orthant. In order to show uniqueness, assume for the sake of contradiction that there exists $y \neq x_e, y \in \mathscr{C}_{x_e}$ such that $\Gamma R(y) = 0$. Then the fundamental theorem of calculus implies,

$$0 = \Gamma R(x_e) - \Gamma R(y) = \Gamma \int_0^1 \frac{\partial R}{\partial x}(tx_e + (1-t)y)\,(x_e - y)dt = \Gamma \frac{\partial R}{\partial x}(x^*)(x_e - y),$$

where $x^* = t^* x_e + (1-t^*)y$, and $t^* \in (0,1)$. The existence of $t^*$ is implied by the integral mean-value theorem. Since $x^* \in \mathscr{C}_{x_e}^{\circ}$, then the reduced Jacobian at $x^*$ is non-singular relative to $\operatorname{Im}\Gamma$. Since $x_e - y \in \operatorname{Im}\Gamma$, then $y = x_e$. This gives a contradiction. □

### 4.1.3 Exponential stability

We have shown that the existence of a PWL RLF function implies that it is a common Lyapunov function for all linear systems that belong to a linear differential inclusion.

In fact, one of the properties of systems that admits a piecewise linear Lyapunov function is that a stable steady state cannot have purely imaginary eigenvalues [11]. Hence, the reduced Jacobian at a non-degenerate steady state cannot admit pure imaginary eigenvalues which implies the following Theorem:

**Theorem A-6.** *Let* $(\mathscr{S}, \mathscr{R})$ *be a network that admits a PWL RLF. If a positive steady state* $x_e$ *is non-degenerate relative to* $\mathscr{C}_{x_e}$*, then it is exponentially asymptotically stable.*

### 4.1.4 Global stability

Establishing global asymptotic stability of a positive steady state for a network that admits a PWL RLF has been accomplished via a LaSalle graphical algorithm in [5]. Nevertheless, if a network is known to be robustly non-degenerate with respect to the stoichiometric class (by the test given in Theorem 7 for instance), then the following result holds:

**Theorem A-7.** *([12]) Suppose that the system* (1) *admits a PWL RLF. If the Jacobian is robustly non-degenerate relative to a stoichiometric class* $\mathscr{C}$*, then every positive steady state* $x_e \in \mathscr{C}$ *is globally asymptotically stable relative to* $\mathscr{C}$*.*

Hence, the graphical LaSalle algorithm is not needed for networks with Jacobians that are robustly non-degenerate relative to stoichiometric classes.

## 4.2 Absence of critical siphons: Proof of Theorem 8

Assume $P$ is a critical siphon for the Petri-net associated with $\Gamma$, and let $n_p = |P|$. Let $\Lambda(P)$ be the set of output reactions of $P$, and let $\nu_p = |\Lambda(P)|$.

Item 1 of Theorem 8 has been proved in [5]. We restate the proof in this paper's terminology for completeness. First, the following lemma is needed.

**Lemma A-2.** *Consider a network $(\mathscr{S},\mathscr{R})$. Let $P$ be a set of species that does not contain the support of a conservation law; let its indices be numbered as $\{1,...,n_p\}$. Then, there exists a nonempty-interior region $\{r|\Sigma_k\Gamma r \geq 0\}$ with a signature matrix $\Sigma_k$ that satisfies $\sigma_{k1} = ... = \sigma_{kn_p} = 1$.*

*Proof.* Assume the contrary. This implies that $\cap_{i=1}^{n_p}\{R|\gamma_i^T R > 0\} \bigcap \cap_{i=n_p+1}^{n}\{R|\sigma_i\gamma_i^T R > 0\} = \emptyset$ for all possible choices of signs $\sigma_i = \pm 1$. However, $\mathbb{R}^r$ can be partitioned into a union of all possible half-spaces of the form $\cap_{i=n_p+1}^{n}\{R|\sigma_i\gamma_i^T R \geq 0\}$. Therefore, this implies that $\cap_{i=1}^{n_p}\{R|\gamma_i^T R > 0\} = \emptyset$. By Farkas Lemma, this implies that there exists $\lambda \in \mathbb{R}^{n_p}$ satisfying $\lambda > 0$ such that $[\lambda^T \mathbf{0}]\Gamma = 0$. Therefore, $P$ contains the support of the conservation law $[\lambda^T\ 0]^T$; a contradiction. □

Therefore, we can state the proof of the first item:

*Proof of Theorem 8-1).* Without loss of generality, let $\{1,...,n_p\}$ be the indices of the species in $P$. Using Lemma 2, there exists a nonempty-interior sign region $\mathcal{S}_k, 1 \leq k \leq m_s$ with a signature matrix $\Sigma_k$ that satisfies $\sigma_{k1} = ... = \sigma_{kn_p} = 1$. Since $\Lambda(P) = \mathscr{R}$, this implies that we must have $c_k \geq 0$ to match the sign pattern of $\Sigma_k$. But since $\exists v \gg 0 \in \ker\Gamma$, then this implies that $c_k = 0$ which contradicts the positive definiteness condition on the RLF since $\ker C \neq \ker\Gamma$. □

In order to proceed, we denote by $\Psi_P$ the *face* that corresponds to a siphon $P$. It is given by: $\Psi_P = \{x \in \mathbb{R}_+^n | X_i \in P \Rightarrow x_i = 0\}$. We state the following lemma next:

**Lemma A-3.** *Consider a network $(\mathscr{S},\mathscr{R})$. Let $P$ be a critical siphon, and let $\Psi_P$ be the associated face. If the network is conservative, then for any proper stoichiometric compatibility $\mathscr{C}$, there exists a steady state $x_e$ of (1) such that $x_e \in \Psi_P \cap \mathscr{C}$.*

*Proof.* The set $\Psi_P \cap \mathscr{C}$ is compact, forward invariant, and convex, since both sets $\Psi_P, \mathscr{C}$ are such. Hence, the statement of the lemma follows directly from the application of the Brouwer Fixed Point Theorem on the associated flow. □

We are ready now to prove the second item of Theorem 8.

*Proof of Theorem 8-2).* By Lemma 3, there exists a steady state in $\Psi_P$. Since it is assumed that there exists a non-degenerate steady state in the interior, Proposition 5 implies that the network cannot admit a PWL RLF. □

Before concluding the proof, a simple lemma is stated and proved:

**Lemma A-4.** *Let $x_e$ be a steady state of (1). Let $\tilde{P}$ be a set of species that correspond to $\{1,..,n\}\backslash\, supp(x_e)$. Then, $\tilde{P}$ is a siphon.*

*Proof.* Assume that $\tilde{P}$ is not a siphon, then there exists some $X_i \in \tilde{P}$ and $\mathbf{R}_j \in \mathscr{R}$ such that $X_i$ is a product of $\mathbf{R}_j$ and $\mathbf{R}_j \neq \Lambda(\tilde{P})$. At the given steady state, all negative terms in the expression of $\dot{x}_i$ vanish since $x_{ei} = 0$. Since $X_i$ is not a reactant in $\mathbf{R}_j$ this implies $\beta_{ij} > 0, \alpha_{ij} = 0$. Therefore, $R_j(x)$ has a strictly positive coefficient, which implies $\dot{x}_i > 0$ resulting in a contradiction. □

Hence, we are ready to conclude the proof of Theorem 8:

*Proof of Theorem 8-3).* By Lemma 3, there exists a steady state $x^* \in \Psi_P$ such that $\Gamma R(x^*) = 0$. Since $\dim(\ker\Gamma) = 1$, this implies that $R(x^*) = tv$ for some $t \geq 0$. Consider the case $t = 0$. This implies $R(x^*) = 0$. Then, $P \subset \tilde{P} := \{1,..,n\}\backslash\,\mathrm{supp}(x^*)$. $\tilde{P}$ is a siphon by Lemma 4, and since $P \subset \tilde{P}$ it is a critical deadlock. However, by Theorem 8-1), the network is does not admit a PWL RLF, which is a contradiction. If $t > 0$, this implies that $P = \emptyset$; giving a contradiction. □

## 5 Concentration-dependent RLFs

### 5.1 Proof of Theorem 9

Let $V(x) = \hat{V}(x - x_e)$. Then at those points $z$ where $\partial\hat{V}/\partial z$ exists, we can write:

$$\dot{V} = \frac{\partial \hat{V}}{\partial z}\dot{z} = \frac{\partial \hat{V}}{\partial z}\sum_{\ell=1}^{s}\rho_\ell(t)\Gamma_{i_\ell}e_{j_\ell}^T z = \sum_{\ell=1}^{s}\rho_\ell(t)\left(\frac{\partial \hat{V}}{\partial z}\Gamma_{i_\ell}e_{j_\ell}^T z\right).$$

Since we have assumed that $\hat{V}$ is a common Lyapunov function for the set of linear systems $\{\dot{z} = (\Gamma_{i_1}e_{j_1}^T)z, ..., \dot{z} = (\Gamma_{i_s}e_{j_s}^T)z\}$, the proof can proceed in both directions in a similar way to the proof of Theorem 1. Notice that the constraint $D^T z = 0$ is needed since $D^T\dot{x}(t) \equiv 0$ is implicit in the structure of the original system (1). $\square$

### 5.2 Proof of Theorem 10

Positive definiteness is clearly satisfied. It remains to show the second condition. Let $z = x - x_e$. Then, whenever $\hat{V}$ is differentiable:

$$\dot{V}_2(x) = \frac{\partial \hat{V}(x - x_e)}{\partial z}\dot{x} = \frac{\partial \hat{V}(x - x_e)}{\partial z}\Gamma R(x).$$

Before proceeding, we prove two statements: First, from (28), we get $(\partial\tilde{V}(r)/\partial r) = (\partial\hat{V}(\Gamma r)/\partial z)\Gamma$. Second, note that $x - x_e \in \mathrm{Im}(\Gamma)$, hence there exists $r \in \mathbb{R}^\nu$ such that $\Gamma r = x - x_e$, where $r$ can always be chosen nonnegative by assumption AS1. Hence, where $\hat{V}$ is differentiable, we can use (27) to write:

$$\begin{aligned}\dot{V}_2(x) &= \frac{\partial \hat{V}(x - x_e)}{\partial r}\Gamma\frac{\partial R(x'')}{\partial x}(x - x_e) = \frac{\partial \tilde{V}(r)}{\partial r}\frac{\partial R(x'')}{\partial x}\Gamma r \\ &= \sum_{\ell=1}^{s}\rho_\ell\frac{\partial \tilde{V}(r)}{\partial r}\Gamma^\ell r \le 0,\end{aligned}$$

where the last inequality follows from (7). Lemma A1 implies that $\dot{V}_2(x) \le 0$ for all $x$. $\square$

### 5.3 Proof of Theorem 11

The first statement follows from Theorem 10. In order to show the second statement, let $V_2(x) = b_k^T(x - x_e)$, for $x - x_e \in \mathcal{V}_k$. We will show that $V_1(x) = c_k^T R(x)$, for each $R(x) \in \mathcal{W}_k$ is nondecreasing along the trajectories. Without loss of generality, the partition matrix can be written in the form: $G = [I\ \hat{G}^T]^T$. This representation implies that the sign of $x - x_e$ is determined in every region $\mathcal{V}_k = \{z|\Sigma_k Gz \ge 0\}$, $k = 1, .., m$, where $\Sigma_k = \mathrm{diag}[\sigma_{k1}, ..., \sigma kn]$ are signature matrices. Now, assume that $x - x_e \in \mathcal{V}_k^\circ$. Then:

$$\dot{V}_2(x) = b_k^T\Gamma R(x) = c_k^T R(x) \le 0 = c_k^T R(x_e), \text{ for all } R \in \mathscr{K}_A.$$

Let $R_j(x) \in \mathrm{supp}\, c_k$, and let $\alpha_{ij} > 0$. Since $R$ is nondecreasing by AK3, if $\mathrm{sgn}(x_i - x_{e_i})\,\mathrm{sgn}(c_{kj}) > 0$, there exists $R \in \mathscr{K}_A$ such that $\dot{V}_2(x) \ge 0$. Hence, this implies that the inequality $\mathrm{sgn}(c_{kj})\,\mathrm{sgn}(x_i - x_{e_i}) \le 0$ holds. Fix $j$. If there exists $i_1, i_2$ such that $\alpha_{i_1 j}, \alpha_{i_2 j} > 0$ and $\mathrm{sgn}(x_{i_1} - x_{e_{i_1}})\,\mathrm{sgn}(x_{i_2} - x_{e_{i_2}}) < 0$, then $\sigma_{kj} := 0$. Otherwise, $\sigma_{kj} := \mathrm{sgn}(x_i - x_{e_i})$ for some $i$ such that $\alpha_{ij} > 0$.
Hence, in order to have $\dot{V}_2(x) \le 0$ for all $R \in \mathscr{K}_A$ we need that $\sigma_{kj}(x_i - x_{e_i}) \ge 0$ whenever

$x - x_e \in \mathcal{V}_k$, for all $k, j, i$ with $\alpha_{ij} > 0$. By Farkas' Lemma [13], this is equivalent to the existence of $\lambda_{kji} \in \bar{\mathbb{R}}^p_+, \zeta_{kji} \in \mathbb{R}^\iota$, such that

$$\sigma_{kj} e_i^T = \lambda_{kji}^T \Sigma_k G + \zeta_{kji}^T D, \tag{15}$$

where $D^T \in \mathbb{R}^{\iota \times n}$ is a matrix whose columns are basis vectors for $\ker \Gamma^T$.
If we multiply both sides of (15) by $\Gamma$ from the left, then we get condition C4 in [5, Theorem 4] which is necessary and sufficient for $\dot{V}_1(x) = \frac{d}{dt}(c_k^T R(x)) \leq 0$. $\square$

# 6 Parameters for Figure 1

For the two mechanisms the total concentrations of the substrate and enzymes are $[X_0]_T = 6, [E]_T = 2.5, [F]_T = 6$. The following ODE has been simulated for the distributive mechanism :

$$\dot{x} = \begin{bmatrix} -1 & 1 & 0 & 0 & 0 & 1 & 0 & 0 & 0 & 0 & 0 & 0 \\ -1 & 1 & 1 & 0 & 0 & 0 & -1 & 1 & 1 & 0 & 0 & 0 \\ 1 & -1 & -1 & 0 & 0 & 0 & 0 & 0 & 0 & 0 & 0 & 0 \\ 0 & 0 & 1 & -1 & 1 & 0 & -1 & 1 & 0 & 0 & 0 & 1 \\ 0 & 0 & 0 & -1 & 1 & 1 & 0 & 0 & 0 & -1 & 1 & 1 \\ 0 & 0 & 0 & 1 & -1 & -1 & 0 & 0 & 0 & 0 & 0 & 0 \\ 0 & 0 & 0 & 0 & 0 & 0 & 1 & -1 & -1 & 0 & 0 & 0 \\ 0 & 0 & 0 & 0 & 0 & 0 & 0 & 0 & 1 & -1 & 1 & 0 \\ 0 & 0 & 0 & 0 & 0 & 0 & 0 & 0 & 0 & 1 & -1 & -1 \end{bmatrix} \begin{bmatrix} 120x_1x_2 \\ 10x_3 \\ 8x_3 \\ 13x_4x_5 \\ 20x_6 \\ 28x_6 \\ 24x_2x_4 \\ 3x_7 \\ \frac{7}{2}x_7 \\ 10x_5x_8 \\ \frac{3}{2}x_9 \\ x_9 \end{bmatrix},$$

where $x_1 = [X_0], x_2 = [E], x_3 = [X_0E], x_4 = [X_1], x_5 = [F], x_6 = [X_1F], x_7 = [X_1E], x_8 = [X_2], x_9 = [X_2F]$.

The following ODE has been simulated for the processive mechanism :

$$\dot{x} = \begin{bmatrix} -1 & 1 & 0 & 0 & 0 & 0 & 0 & 1 \\ -1 & 1 & 0 & 0 & 0 & 0 & 1 & 0 \\ 1 & -1 & -1 & 0 & 0 & 0 & 0 & 0 \\ 0 & 0 & 0 & -1 & 1 & 0 & 1 & 0 \\ 0 & 0 & 0 & -1 & 1 & 0 & 0 & 1 \\ 0 & 0 & 0 & 1 & -1 & -1 & 0 & 0 \\ 0 & 0 & 1 & 0 & 0 & 0 & -1 & 0 \\ 0 & 0 & 0 & 0 & 0 & 1 & 0 & -1 \end{bmatrix} \begin{bmatrix} 120x_1x_2 \\ 2x_3 \\ \frac{8}{5}x_3 \\ \frac{13}{5}x_4x_5 \\ 4x_6 \\ \frac{28}{5}x_6 \\ \frac{24}{5}x_7 \\ \frac{7}{2}x_8 \end{bmatrix},$$

where $x_1 = [X], x_2 = [E], x_3 = [X_1E], x_4 = [X_2], x_5 = [F], x_6 = [X_2F], x_7 = [X_2E], x_8 = [x], x_9 = [X_1F]$.

# 7 The Software Package LEARN

We describe the prerequisites of `LEARN`, the basic subroutines offered and few example runs. `LEARN` can be accessed at `github.com/malirdwi/LEARN`.

## 7.1 Prerequisites

`LEARN` runs on MATLAB with the optimization and symbolic math toolboxes. Also, it needs the `cvx` package. The latest version of `cvx` is available on the link `http://cvxr.com/cvx/download/`. After download, the user must run `cvx_setup`. After `cvx_setup` reporting that `cvx` is successfully installed, `LEARN` should run without issues.

## 7.2 List of Subroutines

The following subroutines are available. Note that all the subroutines below take $\Gamma$ as an input which is the stoichiometry matrix of the network. If the network has an autocatalytic reaction then both matrices $A, B$ need to be entered. (see the Methods section in the main text)

### 7.2.1 Main subroutines

- `LEARNmain(Gamma)`: Prints a basic report on the network. This subroutine should be sufficient for most users. Examples will follow. Another parallel function, `LEARNmainplus(Gamma)`, is available which runs a more exhaustive RLF search.

### 7.2.2 Basic subroutines

- `d=IsConservative(Gamma)`: Checks if the network is conservative. If it is, then the subroutine returns a positive vector $d \in \mathbb{R}_+^n$ such that $d^T\Gamma = 0$. If the network is not conservative then $d$ returns a scalar 0.

- `v=IsAS1(Gamma)`: Checks if the stoichiometry matrix has a positive vector in its kernel. If it does, then the subroutine returns a positive vector $v \in \mathbb{R}_+^n$ such that $\Gamma v = 0$. If the network is not conservative then $d$ returns a scalar 0.

- `[flag,deadlock]=checkSiphons(Gamma)`: Checks if there are critical siphons and deadlocks. Each output can be either 0 or 1.

- `flag=checkMnetwork(Gamma)`: Checks if the network is an $M$-network. The output is either 0 or 1.

### 7.2.3 Necessary Conditions

- `checkSiphonCondition(Gamma)`: Checks if the network violates the critical siphon necessary condition (Theorem 8). It prints a brief report.

- `flag=SignPatternCheck(Gamma)`: Checks if the network violates the sign pattern necessary condition [5, Theorem 9]. The output is either 0 or 1.

- `flag=checkPmatreix(Gamma)`: Checks if the network violates the $P$ matrix necessary condition [5, Theorem 8]. The output is either 0 or 1.

- `flag=RobustNondegeneracy(Gamma)`: Checks if the network has a robustly non-degenerate Jacobian (Theorem 7). This only applies to networks that pass the $P$ matrix test. The output is either 0 or 1.

#### 7.2.4 Construction of RLFs

- `C=ConstructGraphical(Gamma)`: Checks if the network admits the Max-Min RLF as given in Theorem 4. The output is $C$. If the method fails then $C$ will be an empty matrix.

- `C=ConstructIterate(Gamma)`: Checks if the network admits an RLF as given in Theorem 3. The output is $C$. If the method fails then $C$ will be an empty matrix.

- `[C,cvx]=ConstructLP(Gamma,H2,w,c)`: Checks if a non-autocatalytic network admits an RLF as given in Theorem 2. The last three inputs are optional. The output is $C$ and the flag `cvx` to indicate that the RLF has been certified to be convex. The second input is $H_2$ which are optional rows to add to the partitioning matrix $H = \Gamma$. The default value for $H_2$ is an empty matrix. The third input is $w$ and it is a flag to constrain the search to Sum-of-Currents RLFs. The default value is 1, but it is set to 0 in the `LEARNmainplus` subroutine. The fourth input is a flag to constrain the RLF to be convex. The default value is 0 which is the recommended value.

- `[C,cvx]=ConstructLPauto(A,B,H2,w,c)`: Checks if an *autocatalytic* network admits an RLF as given in Theorem 2. The remaining input structure is similar to the previous subroutine.

- `[C]=ConstructCoP(Gamma,H2)`: Checks if a non-autocatalytic network admits an RLF as given in Theorem 5. The last input is optional. The output is a tensor of PWQ RLF matrices. The second input is $H_2$ which are optional rows to add to the partitioning matrix $H = \Gamma$. The default value for $H_2$ is an empty matrix.

#### 7.2.5 Checking a candidate RLF

- `flag=CheckRLF(Gamma,C)`: Checks if $\tilde{V} = \max_k c_k^T r$ is an RLF for a non-autocatalytic network with the stoichiometry matrix $\Gamma$.

### 7.3 Examples

All the examples are included in the folder `examples`.

#### 7.3.1 The double processive PTM cycle

This is the form of the input to `LEARN` for the network depicted in Fig. 9-b.

```
Gamma=[
-1   1   0   0   0   0   0   1;
-1   1   0   0   0   0   1   0;
 1  -1  -1   0   0   0   0   0;
 0   0   0  -1   1   0   1   0;
 0   0   0  -1   1   0   0   1;
 0   0   0   1  -1  -1   0   0;
 0   0   1   0   0   0  -1   0;
 0   0   0   0   0   1   0  -1];
LEARNmain(Gamma)
```

Note that the stoichiometry matrix $\Gamma$ can be easily written from a list of reactions. The output of LEARN is as follows:

```
--------------------------------
Welcome to LEARN v1.01, Jan 2020
Developed by M. Ali Al-Radhawi malirdwi@{northeastern.edu,mit.edu,
   gmail.com}

LEARN tries to construct a Robust Lyapunov Function for a given
   reaction network.
--------------------------------
The network has 8 species and 8 reactions.
The stoichiometric space is 5-dimensional.
The network has a positive vector in the kernel of the stoichiometry
    matrix, i.e. it has the potential for positive steady states.
The network is conservative.
The network has no critical siphons. It is structurally persistent.
--------------------------------
LEARN will check some necessary conditions
Necessary Condition # 1 ....
The critical siphon necessary condition is satisfied.
Necessary Condition # 2 ....
The sign pattern necessary condition is satisfied.
Necessary Condition # 3 ....
The P matrix necessary condition is satisfied.
--------------------------------
LEARN will search for a PWL RLF
Method # 1: Graphical Method ..
This is an M-network. The graphical criteria will be checked

Success!! A PWL RLF has been found.
The following is always a Lyapunov function for any monotone
   kinetics: V(x)=|| C*R(x) ||_infty,
where C is given as follows:
0       0       1       0       0      -1       0       0
0       0       1       0       0       0      -1       0
0       0       1       0       0       0       0      -1
-1      1       1       0       0       0       0       0
0       0       1      -1       1       0       0       0
0       0       0       0       0       1      -1       0
0       0       0       0       0       1       0      -1
-1      1       0       0       0       1       0       0
0       0       0      -1       1       1       0       0
0       0       0       0       0       0       1      -1
-1      1       0       0       0       0       1       0
0       0       0      -1       1       0       1       0
-1      1       0       0       0       0       0       1
0       0       0      -1       1       0       0       1
```

```
1       -1      0       -1      1       0       0       0

The robust non-degeneracy test is passed.
Since the network is conservative and with no critical siphons then
   the following holds:
There exists a unique positive globally asymptotically stable steady
    state  in each stoichiometric class.
--------------------------------
Method # 2: Iterative Method ..
Success!! A PWL RLF has been found.
The following is always a Lyapunov function for any monotone
   kinetics: V(x)=|| C*R(x) ||_infty,
where C is given as follows:
-1      1       0       0       0       0       0       1
-1      1       0       0       0       0       1       0
1       -1      -1      0       0       0       0       0
0       0       0       -1      1       0       1       0
0       0       0       -1      1       0       0       1
0       0       0       1       -1      -1      0       0
0       0       1       0       0       0       -1      0
0       0       0       0       0       1       0       -1
0       0       0       0       0       0       -1      1
0       0       -1      0       0       0       0       1
-1      1       0       0       0       1       0       0
0       0       0       0       0       -1      1       0
0       0       1       -1      1       0       0       0
0       0       -1      0       0       1       0       0
-1      1       0       1       -1      0       0       0

The robust non-degeneracy test is passed.
Since the network is conservative and with no critical siphons then
   the following holds:
There exists a unique positive globally asymptotically stable steady
    state  in each stoichiometric class.
--------------------------------
Method # 3: Linear Programming Method ..
The partition matrix H is set to the default choice H=the
   stoichiometry matrix ..
This method for constructing a PWL RLF has failed.
THE END.
```

### 7.3.2 The double distributive PTM cycle

This is the output of `LEARNmain` for the network depicted in Fig. 9-d.

```
--------------------------------
Welcome to LEARN v1.01, Jan 2020
```

```
?Developed by M. Ali Al-Radhawi malirdwi@{northeastern.edu,mit.edu,
   gmail.com}

LEARN tries to construct a Robust Lyapunov Function for a given
   reaction network.
--------------------------------
The network has 9 species and 12 reactions.
The stoichiometric space is 6-dimensional.
The network has a positive vector in the kernel of the stoichiometry
    matrix, i.e. it has the potential for positive steady states.
The network is conservative.
The network has no critical siphons. It is structurally persistent.
--------------------------------
LEARN will check some necessary conditions
Necessary Condition # 1 ....
The critical siphon necessary condition is satisfied.
Necessary Condition # 2 ....
The sign pattern necessary condition is satisfied.
Necessary Condition # 3 ....
The P matrix necessary condition is violated. A PWL RLF does not
   exist
--------------------------------
LEARN will search for a PWL RLF
Method # 1: Graphical Method ..
This is not an M-network. Method # 1 is not applicable.

--------------------------------
Method # 2: Iterative Method ..
This method for constructing a PWL RLF has failed.
--------------------------------
Method # 3: Linear Programming Method ..
The partition matrix H is set to the default choice H=the
   stoichiometry matrix ..
This method for constructing a PWL RLF has failed.
THE END.
```

#### 7.3.3 The McKeithan Network

This is the output of `LEARNmain` for the network depicted in Fig. 11-a with $N = 2$.

```
--------------------------------
Welcome to LEARN v1.01, Jan 2020
Developed by M. Ali Al-Radhawi malirdwi@{northeastern.edu,mit.edu,
   gmail.com}

LEARN tries to construct a Robust Lyapunov Function for a given
   reaction network.
--------------------------------
```

```
The network has 5 species and 6 reactions.
The stoichiometric space is 3-dimensional.
The network has a positive vector in the kernel of the stoichiometry
    matrix, i.e. it has the potential for positive steady states.
The network is conservative.
The network has no critical siphons. It is structurally persistent.
--------------------------------
LEARN will check some necessary conditions
Necessary Condition # 1 ....
The critical siphon necessary condition is satisfied.
Necessary Condition # 2 ....
The sign pattern necessary condition is satisfied.
Necessary Condition # 3 ....
The P matrix necessary condition is satisfied.
--------------------------------
LEARN will search for a PWL RLF
Method # 1: Graphical Method ..
This is not an M-network. Method # 1 is not applicable.

--------------------------------
Method # 2: Iterative Method ..
Success!! A PWL RLF has been found.
The following is always a Lyapunov function for any monotone
   kinetics: V(x)=|| C*R(x) ||_infty,
where C is given as follows:
-1      1       0       0       1       1
-1      1       0       0       1       1
1       -1      -1      0       0       0
0       0       1       -1      -1      0
0       0       0       1       0       -1
0       0       -1      0       1       1
-1      1       1       -1      0       1
-1      1       0       1       1       0

The robust non-degeneracy test is passed.
Since the network is conservative and with no critical siphons then
   the following holds:
There exists a unique positive globally asymptotically stable steady
    state  in each stoichiometric class.
--------------------------------
Method # 3: Linear Programming Method ..
The partition matrix H is set to the default choice H=the
   stoichiometry matrix ..
Success!! A PWL RLF has been found.
The following is always a Lyapunov function for any monotone
   kinetics: V(x)=|| C*R(x) ||_infty,
where C is given as follows:
0       0       0       1       0       -1
```

```
0         0        1       -1       -1        0
0         0        1        -0      -1       -1
1        -1       -1        0        0        0
1        -1       -1        1        0       -1
1        -1        -0      -1       -1        0
1        -1        -0       -0      -1       -1

The robust non-degeneracy test is passed.
Since the network is conservative and with no critical siphons then
   the following holds:
There exists a unique positive globally asymptotically stable steady
    state  in each stoichiometric class.
Please note that this function is a Sum-of-Currents RLF
which can alternatively be written
as V(x)= sum_i xi_i |dot x_i|, where xi=[xi_1 .... xi_n]=
 1        1        2        2        2
THE END.
```

## References


1. W. Hahn. *Stability of Motion.* Springer-Verlag, New York, 1967.
2. T. Yoshizawa. *Stability theory by Liapunov's Second Method.* Mathematical Society of Japan, Tokyo, 1966.
3. F. H. Clarke, Y. Ledyaev, R. Stern, and P. Wolenski. *Nonsmooth Analysis and Control Theory.* Springer, New York, 1997.
4. M. Feinberg. Chemical reaction network structure and the stability of complex isothermal reactors–I. The deficiency zero and deficiency one theorems. *Chemical Engineering Science*, 42(10):2229–2268, 1987.
5. M. Ali Al-Radhawi and David Angeli. New approach to the stability of chemical reaction networks: Piecewise linear in rates lyapunov functions. *IEEE Transactions on Automatic Control*, 61(1):76–89, 2016.
6. B. Bereanu. A property of convex piecewise linear functions with applications to mathematical programming. *Mathematical Methods of Operations Research*, 9(2):112–119, 1965.
7. A. Polanski. On infinity norms as Lyapunov functions for linear systems. *IEEE Transactions on Automatic Control*, 40(7):1270–1274, 1995.
8. A. P. Molchanov and E. S. Pyatnitskii. Lyapunov functions that specify necessary and sufficient conditions of absolute stability of nonlinear nonstationary control systems. I,III. *Automation and Remote Control*, 47:344–354, 620–630, 1986.
9. H. Kiendl, J. Adamy, and P. Stelzner. Vector norms as Lyapunov functions for linear systems. *IEEE Transactions on Automatic Control*, 37(6):839–842, 1992.
10. M. Banaji, P. Donnell, and S. Baigent. P matrix properties, injectivity, and stability in chemical reaction systems. *SIAM Journal on Applied Mathematics*, 67(6):1523–1547, 2007.
11. E. B Castelan and J.-C. Hennet. Eigenstructure assignment for state constrained linear continuous time systems. *Automatica*, 28(3):605–611, 1992.
12. F. Blanchini and G. Giordano. Polyhedral lyapunov functions structurally ensure global asymptotic stability of dynamical networks iff the jacobian is non-singular. *Automatica*, 86:183–191, 2017.
13. R. T. Rockafellar. *Convex Analysis.* Princeton University Press, New Jersey, 1970.
